# Cost-effectiveness analysis of an implantable cardiac defibrillator compared with pharmaceutical therapy in patients hospitalized with arrhythmia in Iran

**DOI:** 10.1186/s12962-025-00661-5

**Published:** 2025-10-15

**Authors:** Tayyebeh Nowruzi, Reza Goudarzi, Maryam Aliramezany, Mohammadreza Harandi Moghadam, Gholamreza Bazmandegan, Saman Najafi

**Affiliations:** 1 Health Economics, School of Nursing and Midwifery, Rafsanjan University of Medical Sciences, Rafsanjan, Iran; 2 Health Services Management Research Center, Institute for Future Studies in Health, Kerman University of Medical Sciences, Kerman, Iran; 3 Cardiovascular Research Center, Institute of Basic and Clinical Physiology Sciences, Kerman University of Medical Sciences, Kerman, Iran; 4 Rafsanjan Universities of Medical Sciences, Rafsanjan, Iran; 5 Department of Physiology and Pharmacology, School of Medicine, Rafsanjan University of Medical Sciences, Rafsanjan, Iran; 6 Social Determinants of Health Research Center, Yasuj University of Medical Sciences, Yasuj, Iran

**Keywords:** Cardiac arrhythmia, Implantable cardiac defibrillator, Pharmaceutical therapy, Cost-effectiveness

## Abstract

Arrhythmia is a type of cardiovascular disorder that can be fatal. Several methods for treating cardiac arrhythmia have been developed, the most significant of which are implantable cardiac defibrillator (ICD) placement and pharmaceutical therapy. The present study aimed to analyze the cost-effectiveness of an ICD strategy compared to the pharmaceutical therapy in patients with arrhythmia in Iran.This is an economic evaluation study that was carried out using the Markov model for lifetime time horizon. The study population includes all patients with cardiac arrhythmia hospitalized in Shafa hospital in Kerman, eastern-south of Iran, between 2017 and 2019. The study sample includes 101 patients who were treated using the ICD method and 101 randomly selected patients who were received pharmaceutical treatment. This study is conducted from the perspective of the health system, and only direct costs (medical and non-medical) are derived. The study’s outcome was defined as the quality-adjusted life-year (QALY). The data was analyzed using TreeAge software version 2020, and the incremental cost-effectiveness ratio (ICER) was compared to a willingness-to-pay (WTP) threshold of three times GDP per capita in 2021 ($13,002). Deterministic and probabilistic sensitivity analyses were performed to examine the effect of uncertain model parameters on cost effectiveness anlysis. The results revealed that the lifetime costs of ICD and pharmaceutical therapy strategies in treating cardiac arrhythmia were $41,135 and $8,592, respectively, with outcomes equal to 12.63 and 11.29 QALYs. The ICER of the ICD strategy compared to the pharmaceutical therapy was $24,286 per QALY, and it was not cost-effective. The probabilistic sensitivity analysis demonstrated that at a WTP of three times the GDP per capita, the ICD approach had a 40% cost-effectiveness probability. According to the findings of this study, the ICD strategy, when compared to pharmaceutical therapy, is not cost-effective for the treatment of patients with cardiac arrhythmia in Iran. Resource allocation and cost-cutting measures must be implemented as needed.

## Background

Cardiovascular diseases (CVDs) are chronic non-communicable diseases that cause high mortality worldwide and have been an enormous social burden in many nations for many years [1]. The term non-communicable diseases refer to a set of conditions that are not essentially caused by an acute infection, have long-term health-related effects, and generally require long-term care and treatment [2]. Cardiac diseases are caused by the heart’s inability to effectively circulate blood throughout the body, putting patients’ lives at risk [3].

Sudden cardiac death refers to the unexpected natural death within a period of less than one hour from symptoms onset due to cardiac abnormalities in an individual without any prior history of cardiac disease. In the US, more than 350,000 sudden cardiac deaths occur annually. Although accurate statistics of sudden cardiac death are not available for Iran, overall, its increasing trend has been reported in the studies [4]. According to the statistics, cardiovascular diseases account for 46% of all deaths [5].

The majority of sudden and unexpected deaths are caused by cardiac arrhythmia [6]. Cardiac arrhythmia is defined as an abnormality in the heart rhythm with various mechanisms, including automaticity, triggered activity and reentry. Several methods have been applied for their treatment, such as lifestyle modification, pharmaceutical therapy, surgery, implantation of pacemakers or ICDs, and ablation [7].

In the pharmaceutical therapy approach, drugs are categorized based on their electrophysiological effects on normal Purkinje fibers in vitro. Anti-arrhythmic drugs are used for the treatment of heartbeat irregularity. According to the types, severity, and mechanisms of arrhythmias, medications for arrhythmia treatment can be classified into five drug classes, including sodium channel blockers, sympathetic blockers (specifically, β-blockers), potassium channel inhibitors, calcium channel inhibitors, and miscellaneous agents not categorized as above (such as Digoxin and Adenosine) [8, 9].

An implantable cardiac defibrillator (ICD) is an electrical generator placed subcutaneously in the chest and controls the heartbeat and rhythm through its leads. In addition to considerable effects in controlling dysrhythmia, the prediction of arrhythmia occurrence by the implantable defibrillator can highly reduce the risk of sudden cardiac death and contribute to an increased lifetime of the patients while improving their life quality [10].

Numerous studies have shown that the costs and outcomes of implantable defibrillator intervention are far more than those of the pharmaceutical therapy [11–13]. Given the remarkable advancements in technology and the emergence of new drugs and services, right and appropriate decision-making is often challenging for health system policymakers. In addition to its effectiveness, the costs of the new intervention are also of utmost significance because the health systems are faced with resource limitations and have to exploit the existing resources optimally [14].

Economic evaluation is a method that is used to assist policymakers. For the economic evaluation of various interventions, a number of approaches are available, one of which is cost-effectiveness analysis. In this method, all costs and outcomes of two or more interventions are collected and compared against each other, which ultimately results in the maximum achievement that policymakers seek [15].

Based on these considerations and given the lack of economic evaluation studies focusing on treatment interventions for cardiac arrhythmia in Iran, the present study aims to explore the cost-effectiveness of these interventions in patients diagnosed with arrhythmia.

## Methods

### Sample and sample frame

The study population included all patients with cardiac arrhythmia who were admitted to Kerman’s Shafa hospital, southeastern Iran, between 2017 and 2019. The study sample included all 101 ICD patients and 101 pharmaceutical therapy patients who were matched with ICD patients based on age, gender, and clinical conditions. The study included all hospitalized patients who were treated with the ICD method. Patients in the pharmaceutical therapy group, on the other hand, were chosen using simple random sampling based on their medical record number, with matching taken into account. The present study was approved by the Research Committee of Kerman University of Medical Sciences.

### Interventions

In this study, ICD and pharmaceutical therapy strategies are used. These two interventions were chosen for cardiac arrhythmia treatment because they can be used interchangeably and have been studied and compared in numerous studies around the world. Since some patients are sensitive to pharmaceutical therapy, ICD implantation may be preferable. Furthermore, high-risk patients were given ICD from the beginning.

### Perspective and cost

This study is based on the health-care system’s perspective and only takes into account direct (medical and non-medical) costs. The researchers held several expert panel with the cardiac group of Kerman’s Shafa hospital to specify each patient’s health state in ICD and pharmaceutical therapy groups. The researchers then gathered cost data from the medical records of 202 arrhythmic patients treated with either ICD (*n* = 101) or pharmaceutical therapy (*n* = 101). Costs of drugs (in the pharmaceutical therapy group), testing, physician visits, and diagnostic services were extracted from medical records of patients admitted to Kerman’s Shafa hospital between 2017 and 2019. All costs were converted to US dollars using the Central Bank of the Islamic Republic of Iran’s 2021 conversion rate (1 USD = 42000 Rials) [16].

### Outcome

The outcome in this study was measured in Quality-Adjusted Life-Years (QALYs), and utility values for different health states were derived from various studies (Table 1).

**Table 1:** The input data for the Markov model

Model inputs	ICD		Pharmaceutical therapy		Distribution	Reference
	Mean	SD	Mean	SD		
**Transition probabilities**						
Recovery after hospitalization	0.822	-	0.716	-	-	[17]
Hospitalization to death (in ICD)	0.178	0.008	0.284	0.009	beta	[17]
Complication to the well state	0.768	-	-	-	-	[17]
Complication to ICD rejection	0.022	-	-	-	-	[17]
Complication to death	0.21	0.109	-	-	beta	[17]
Recovery after the ICD rejection	0.822	-	-	-	-	[19, 25]
ICD Rejection to death	0.178	0.008	-	-	beta	[19, 25]
**Costs ($)**						
Well	3398	1105	741	1246	gamma	
Complication	3221	933	-	-	gamma	
Hospitalization	2925	1247	1465	2024	gamma	
Death	2777	-	467	-	-	
**Outcome (utility states)**						
Well	0.845	0.124	0.88	0.10	beta	[20–25]
Complication (ICD)	0.75	-	-	-	-	[20–22]
Hospitalization	0.725	-	0.85	-	-	[21]

Transition probabilities between different states of cardiac arrhythmia disease were extracted from other countries’ studies due to the lack of such information in Iran (Table 1). All costs and outcomes in this study were adjusted using a discount rate of 0.06 (range: 0.03–0.09) [18].

### Model overview

**Fig. 1:**
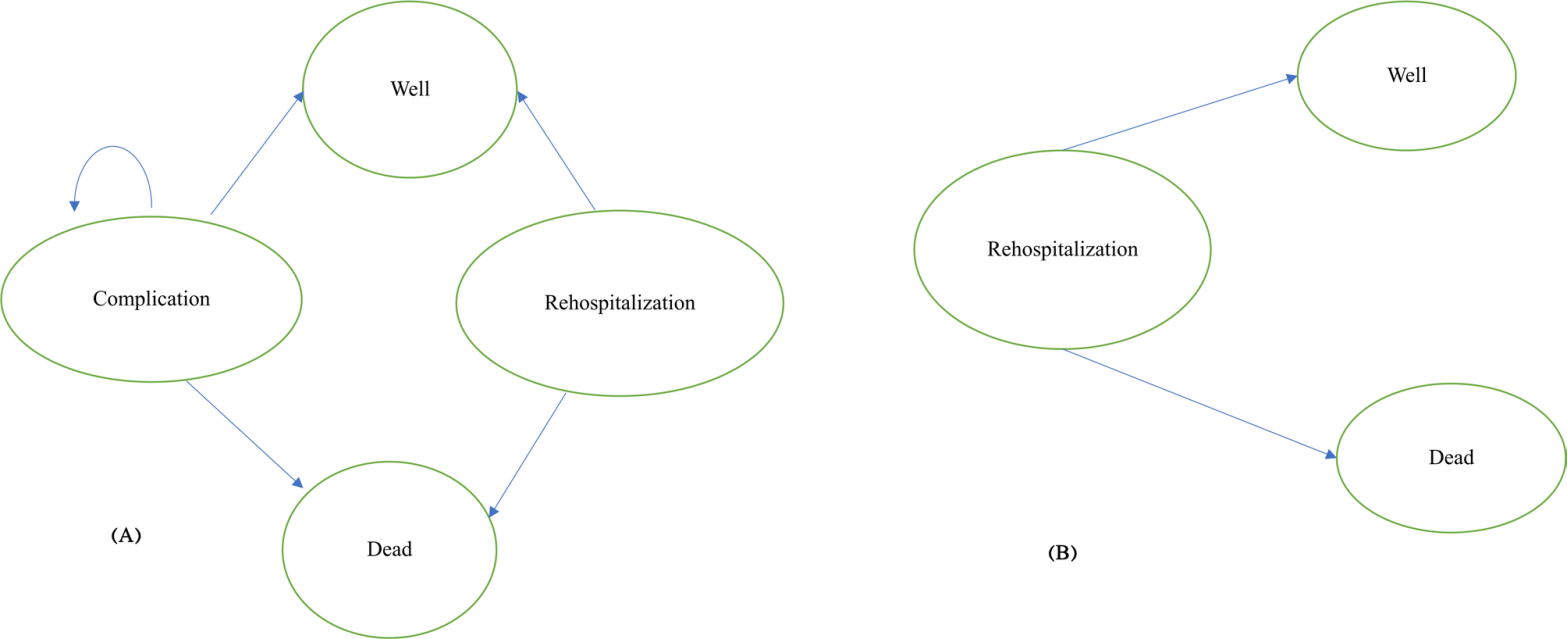
The Markov model of cardiac arrhythmia disease. **A**: Markov model states of disease with ICD; **B**: Markov model states of disease with pharmaceutical therapy

Given the chronic nature of the disease, the Markov decision analysis model was used. Based on the research team’s experience and a similar study, this model was developed [13]. Markov’s states included “well,” “hospitalization,” “complication,” and “death,” which represent the states of patients suffering from cardiac arrhythmia (Fig. 1). Patients that underwent treatment with the ICD strategy faced four states: They either recovered, developed complications, were re-hospitalized for various reasons, or died. The patients with complications either recovered or had a reaction to the device and rejected it or died. Patients that experienced the device rejection either reached a stable state and recovered or died. The re-hospitalized patients either recovered or died. Conversely, only three states were encountered in patients treated with the pharmaceutical therapy: they either recovered, were re-hospitalized, or died. The re-hospitalized patients experienced similar states to the ICD group. The duration of each Markov cycle was one year, and the horizon time of the study was considered the patients’ lifetime.

### Cost-effectiveness analysis

The Markov model was run using TreeAge software version 2020. Incremental cost-effectiveness ratio (ICER) of ICD and pharmaceutical therapy interventions compared with the willingness-to-pay (WTP) threshold of three times the gross domestic production (GDP) per capita, which was equal to $13,002 in 2021 [17]. In this study, the costs were discounted with a 6% rate and outcomes with a 3% rate [18]. The following formula was used to calculate the ICER:

ICER = (Cost_ICD_ – Cost _Pharmaceutical therapy_)/(Effect_ICD_ – Effect _Pharmaceutical therapy_).

### Sensitivity analysis

Both deterministic and probabilistic sensitivity analyses were performed to explore uncertainties. In deterministic sensitivity analysis, initially, all model parameters, including costs, utility values, transition probabilities, and discount rates, were defined in TreeAge software based on a confidence interval of 95%. The Tornado diagram was then applied to specify the most influential parameters on cost-effectiveness results. In probabilistic sensitivity analysis, the parameters with statistical distribution were defined based on statistical distribution type, and using the Monte Carlo simulation were performed with 1000 iterations then the final analysis was carried out using the cost-effectiveness acceptability curve and Incremental cost-effectiveness ratio (ICER) scatterplot.

### Role of the funding source

The funder had no role in study design, data collection, data analysis, interpretation of the study results, writing of the report, or the decision to submit the manuscript for publication.

## Results

This study explored the data of 202 patients treated for cardiac arrhythmia in Shafa Hospital in Kerman between 2017 and 2019. The ICD strategy was used on 101 patients, while pharmaceutical therapy was used on the other 101. The average age of patients was 57 years in the ICD group and 58 years in the pharmaceutical therapy group. There was a male predominance, with men accounting for 79% of cases in the ICD and 78% in the pharmaceutical therapy strategies (Table 2).

**Table 2:** Demographic variables of patients with cardiac arrhythmia hospitalized in Shafa hospital in Kerman

Parameter		Strategy		Total
		ICD	Pharmaceutical therapy	
Age groups	7–18	1	1	2
	19–30	4	4	8
	31–59	45	45	90
	> 60	51	51	102
Gender	Female	21	22	43
	Male	80	79	159

### Base case analysis

According to our findings, the lifetime costs of ICD and pharmaceutical therapy strategies for treating cardiac arrhythmia were $41,135 and $8,592, respectively. In terms of outcomes, ICD and pharmaceutical therapy were associated with 12.63 and 11.29 QALYs, respectively. The ICER for ICD compared to pharmaceutical therapy was $24,286 per QALY, which was much higher than the WTP threshold of three times the GDP ($13,002 per QALY) (Table 3). This result indicates that compared to the pharmaceutical therapy, the ICD strategy is not cost-effective and it is located in the northeastern region of the cost-effectiveness plane and above the WTP threshold line.

**Table 3:** Cost-effectiveness of ICD compared to pharmaceutical therapy in treating patients with cardiac arrhythmia in Iran

Strategy	QALY	Incremental QALY	Cost ($)	Incremental cost ($)	ICER ($/QALY)	ACER	NMB($)
ICD	12.63	1.35	41,135	32,543	24,286	3,257	169,155
Pharmaceutical therapy	11.29	-	8,592	-	-	761	179,387

**Table 4:** Results of cost-effectiveness of different countries

Authors	Country	Incremental cost	Incremental effect	ICER	Result
Atehortúa et al. [13]	Colombia	10,018	0.7596	13,187	Cost-effective
Cowie et al. [21]	Europe	46,413	1.57	29,530	Cost-effective
Sanders et al. [11]	USA	92,100	2.64	34,886	Cost-effective
Ribeiro et al. [19]	Brazil	46,222	0.92	50,345	Not cost-effective
Sanders et al. [20]	USA	69,620	0.5028	138،464	Not cost-effective
Study	Iran	32,543	1.35	24,286	Not cost-effective

### Sensitivity analysis

**Fig. 2:**
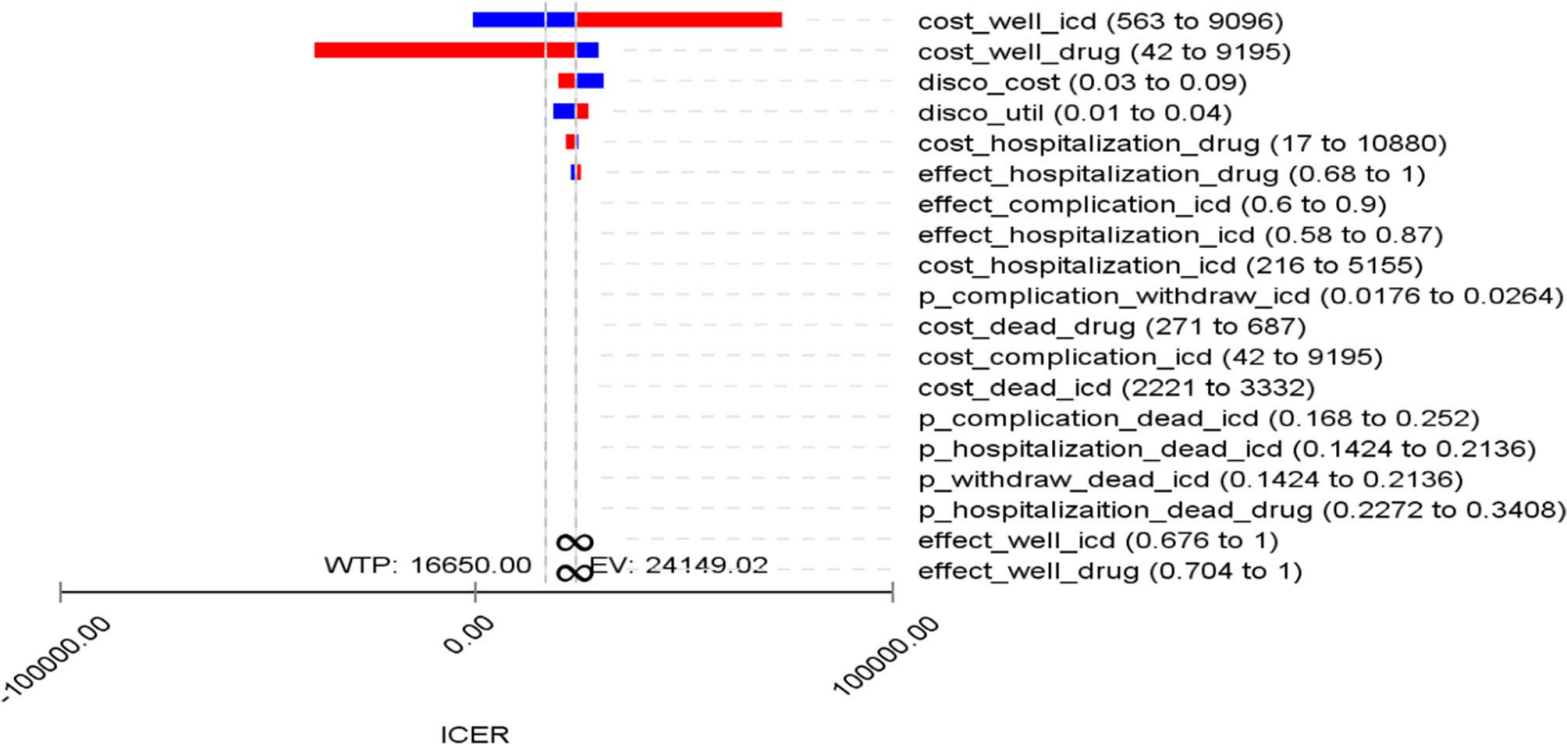
Tornado plot for ICD and pharmaceutical therapy strategies

The Tornado diagram presents the impact of individual input parameters on the ICER per QALY (Fig. 2). As it is shown, the six variables that had the greatest effect on ICER in descending order were: the cost of state of well in ICD, the cost of state of well in pharmaceutical therapy, cost and utility discount rates, the cost of hospitalization for pharmaceutical therapy, and utility of hospitalization in pharmaceutical therapy.

**Fig. 3:**
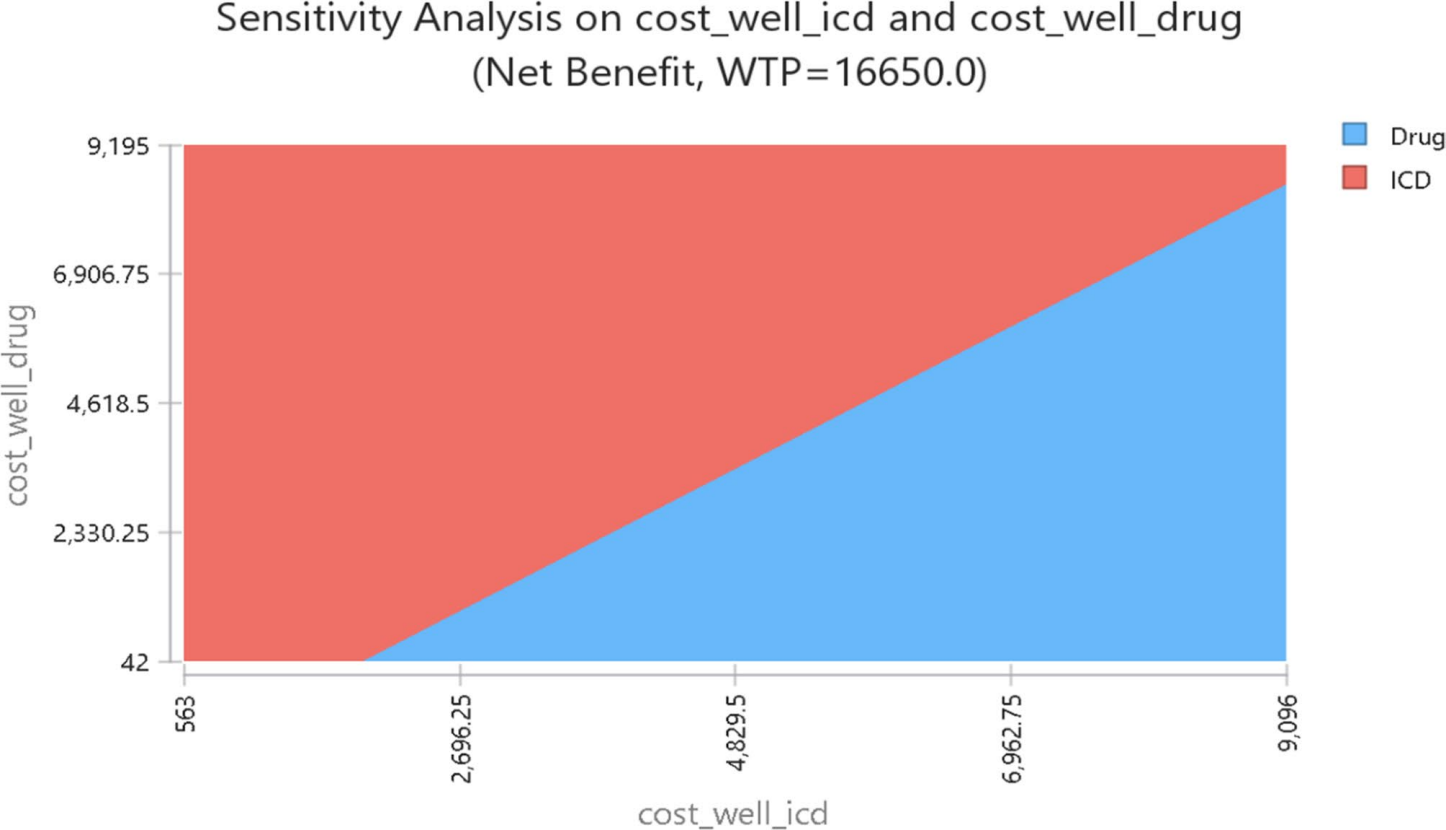
Two-way sensitivity analysis of the cost of state of well in ICD and pharmaceutical therapy strategies

Figure 3 shows the two-way sensitivity analysis results varying the cost of state of well in pharmaceutical therapy ranging from $42 to $9195 and the cost of state of well in ICD ranging $563 to $9096. In combinations of low cost of well state in both ICD and pharmaceutical therapy, ICD was favored.

**Fig. 4:**
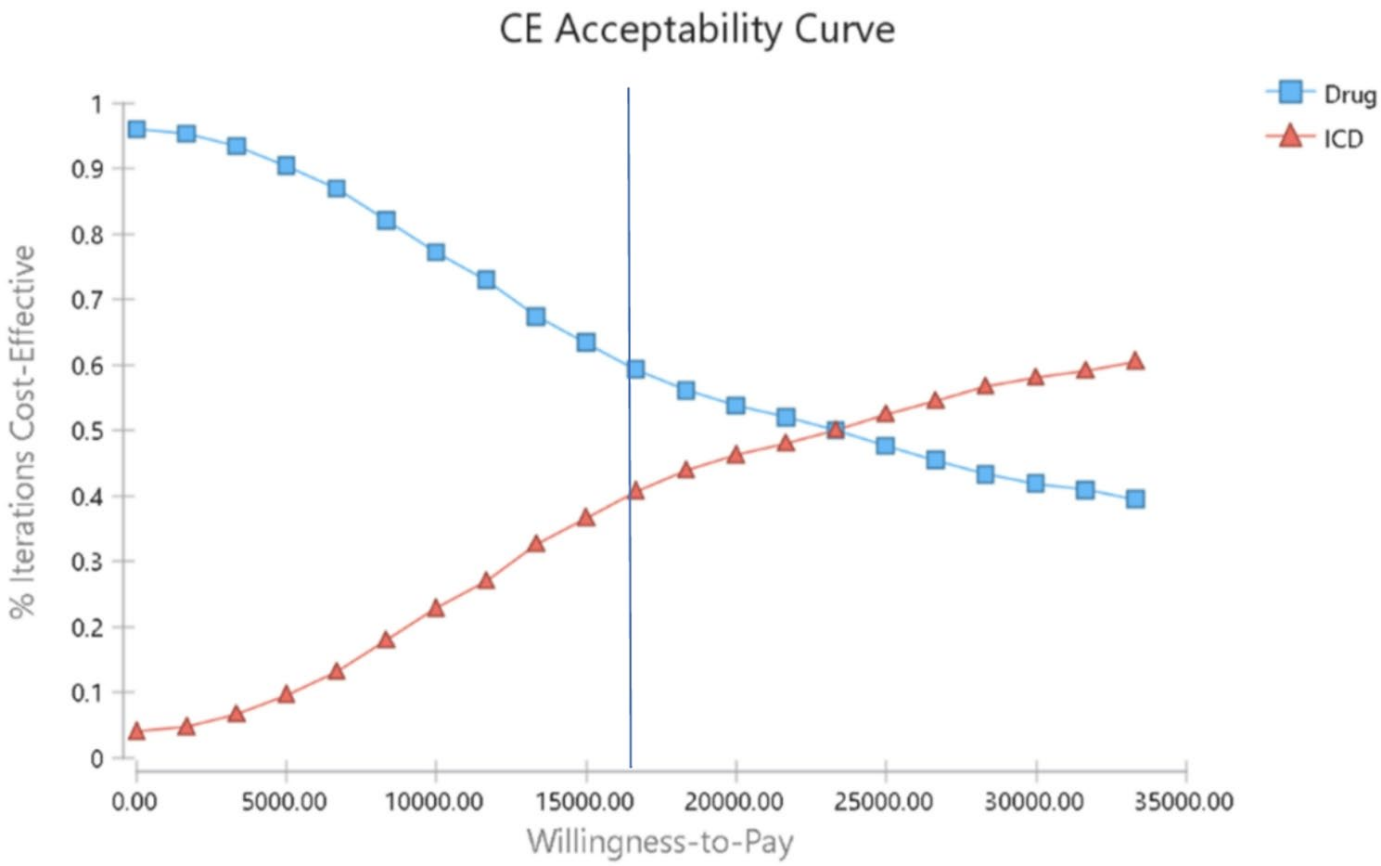
Cost-effectiveness acceptability curve ICD v. pharmaceutical therapy

**Fig. 5:**
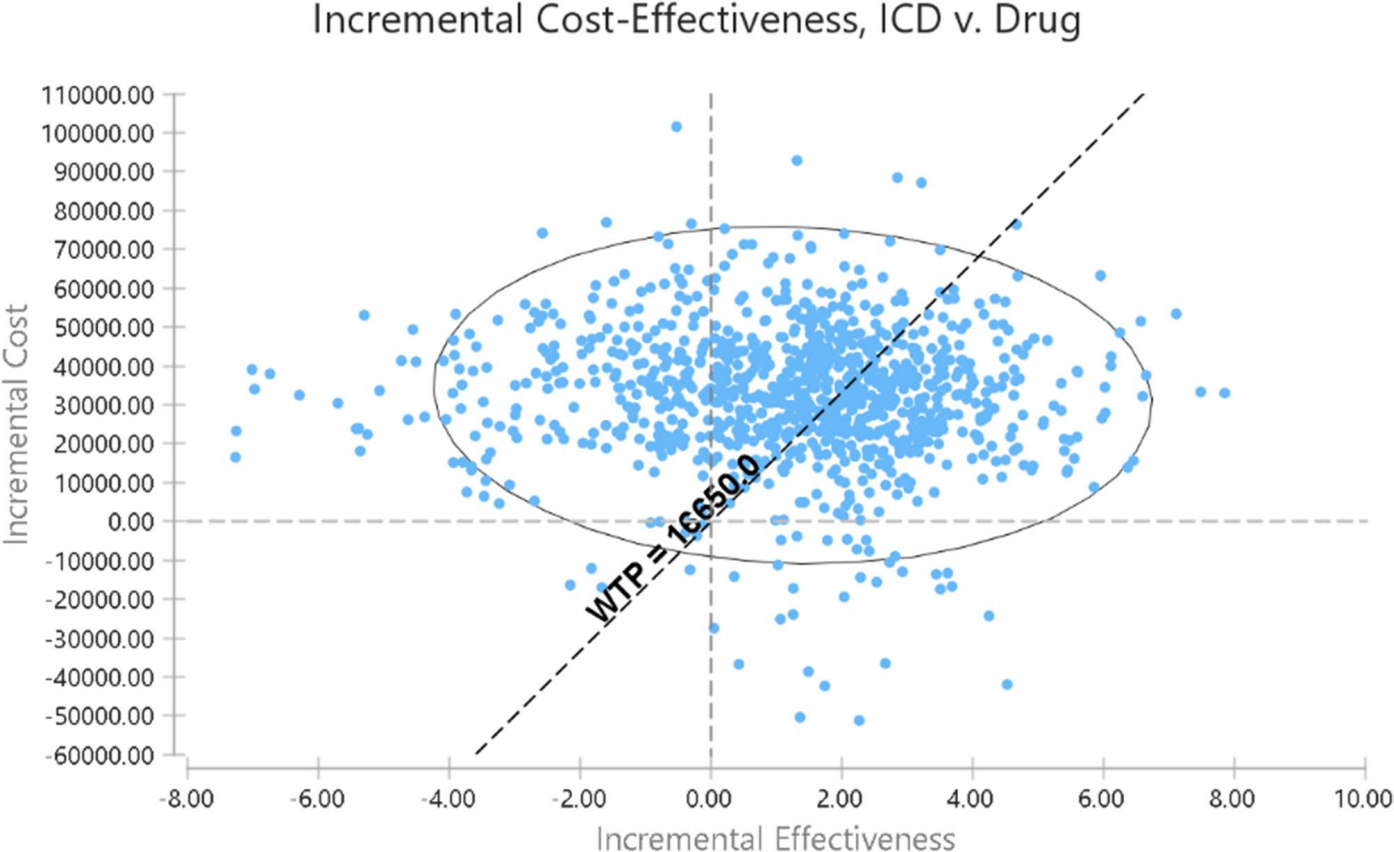
Incremental cost-effectiveness ratio (ICER) scatterplot for ICD compared to pharmaceutical therapy

Plots 4 and 5 illustrate the results of probabilistic sensitivity analysis. As shown in Fig. 4, the cost-effectiveness probability of ICD strategy at a WTP threshold (of three times the GDP per capita) is equal to 40%, which suggests that ICD strategy is not cost-effective in the considered threshold (Figs. 4 and 5). In this figures, the term “drug” refers to pharmaceutical therapy.

## Discussion

Implantable cardioverter-defibrillator (ICD) implantation is widely recognized as one of the most effective interventions for cardiac arrhythmia, providing substantial benefits in arrhythmia management, prolonging survival, and improving quality of life [19, 20]. In this study, we evaluated the cost-effectiveness of ICD therapy compared with pharmaceutical therapy using a lifetime Markov model.

Our findings indicated that the lifetime costs for ICD therapy were USD 41,135, whereas patients treated with pharmaceutical therapy incurred USD 8,592. Thus, ICD therapy imposed nearly 4.8 times higher costs on the health system. Similar results have been reported in international studies. For example, Sanders et al. estimated ICD costs at USD 130,880 versus USD 61,261 for pharmaceutical therapy over a 14-year horizon [11]. In another analysis, Sanders et al. reported ICD costs of USD 130,400 compared with USD 38,300 for pharmaceutical therapy, corresponding to a 3.4-fold increase [11]. Ribeiro et al. in Brazil reported costs of USD 70,841 for ICDs and USD 24,619 for pharmaceutical therapy over 20 years, indicating a 2.9-fold difference [23]. Gandjour et al. in Germany found costs of €101,860 for ICD therapy compared with €56,280 for pharmaceutical therapy, representing a 1.8-fold increase [12]. Atehortúa et al. in Colombia estimated costs of USD 10,992 for ICD plus pharmaceutical therapy and USD 974 for pharmaceutical therapy alone, showing an 11.3-fold increase [13]. Collectively, these findings are consistent with our results and confirm that ICD implantation represents a considerably more expensive treatment strategy across countries.

In terms of outcomes, our study estimated 12.63 QALYs for ICD therapy and 11.29 QALYs for pharmaceutical therapy. These results are in agreement with previous studies, including those by Sanders et al. [11], Gandjour et al. [12], Ribeiro et al. [23], and Cowie et al. [24], which also demonstrated improved health outcomes with ICDs compared with pharmaceutical therapy (Table 4).

Economic evaluation is ultimately judged by the incremental cost-effectiveness ratio (ICER). In our analysis, the ICER was USD 24,286 per QALY, which exceeded the willingness-to-pay (WTP) threshold of USD 13,002 per QALY, indicating that ICD therapy is not cost-effective under current conditions. These findings contrast with some reports in the literature. For instance, Atehortúa et al. [13] reported an ICER of USD 13,187 per QALY in Colombia, below the WTP threshold of USD 19,139 per QALY, indicating cost-effectiveness. Similarly, Cowie et al. [24] reported an ICER of €29,530 per QALY in Europe, and Sanders et al. found ICD therapy cost-effective in U.S. analyses [11, 21].

The discrepancy between our findings and those of other countries can be attributed to two key factors: (i) in Iran, the incremental cost difference between ICD and pharmaceutical therapy is large, and (ii) the incremental QALY gain is relatively modest. Inflationary pressures and international sanctions may further increase ICD-related expenditures, reducing cost-effectiveness in the Iranian health system. Nonetheless, not all international evidence contradicts our results. Ribeiro et al. [23] in Brazil reported an ICER of USD 50,345 per QALY, far above the threshold of three times GDP per capita, concluding that ICD therapy was not cost-effective in that setting. Likewise, Sanders et al. [21] estimated an ICER of USD 138,464 per QALY for ICDs in patients over 65 years of age in the U.S., showing that ICD therapy may not be cost-effective in certain subgroups.

In interpreting these cross-country differences, it is important to recognize factors beyond general economic context. Variation in study design, such as the chosen time horizon, model structure, and analytical perspective, can substantially influence cost-effectiveness outcomes. Differences in patient populations—for example, whether ICDs were evaluated for primary versus secondary prevention or among older versus younger cohorts—also contribute to divergent findings. Moreover, WTP thresholds vary widely across countries, reflecting differences in GDP per capita and national health priorities. Finally, structural features of health systems, including procurement processes, device pricing policies, and the costs of follow-up and hospital care, play a critical role in shaping ICER estimates. Taken together, these methodological and contextual differences help explain why ICD therapy may be deemed cost-effective in some settings but not in others.

Our one-way sensitivity analysis demonstrated that the costs associated with the “well” state in both ICD and pharmaceutical therapy were the most influential parameters on cost-effectiveness. In comparison, Gandjour et al. [12] identified the ICD hazard ratio as most influential, Atehortúa et al. [13] emphasized the mortality probabilities of ICD versus pharmaceutical therapy, and Sanders et al. [22] highlighted utility values for ICD therapy. These differences underscore the importance of context-specific parameters in shaping the economic value of ICD therapy.

This study has some limitations that should be acknowledged. First, transition probabilities were derived from international studies because local data were not available in Iran. Similarly, due to the absence of studies measuring utility values in the Iranian context, utility estimates were also obtained from international sources. Second, the inclusion of direct non-medical costs was incomplete. Although some patient welfare expenditures, such as accommodation services documented in patient files, were extracted, these costs were not reported separately because the primary objective was not comprehensive cost accounting. In addition, certain non-medical costs, such as transportation and lodging, were not available and therefore were not included in the analysis. Third, background all-cause mortality was not incorporated into the Markov model, as age-specific mortality unrelated to cardiac arrhythmia could not be integrated with the available data. Consequently, the “death” state in the model primarily reflected disease progression or intervention-related complications. While this approach is consistent with several previous ICD cost-effectiveness models [11–13, 22, 23], the omission of background mortality may reduce the realism of lifetime projections. Finally, the unfavorable ICER observed in this study was strongly influenced by the use of the official exchange rate and the lack of an explicitly endorsed WTP threshold in Iran. Under alternative assumptions, such as applying a market-based exchange rate or adopting an official WTP threshold, ICD therapy could potentially become cost-effective.

## Conclusion

Despite the study finding that the ICD intervention is not cost-effective compared to pharmaceutical therapy among patients with cardiac arrhythmia, it plays an essential role in treating and rapidly suppressing arrhythmia in patients with heart failure and can prevent sudden death due to arrhythmia. ICDs play a fundamental role in preventing deaths, and it is highly valuable even if it prevents only one death, so it is recommended to implement strategies to reduce ICD costs.

## Data Availability

The datasets used and/or analyzed during the current study are available from the corresponding author on reasonable request.

## Abbreviations

ICD: Implantable Cardiac Defibrillator
ICER: Incremental Cost Effectiveness Ratio
WTP: Willingness To Pay
QALY: Quality Adjusted Life Year
CVD: Cardiovascular diseases
